# Study protocol - efficacy of an attachment-based working alliance in the multimodal pain treatment

**DOI:** 10.1186/s40359-016-0114-7

**Published:** 2016-02-16

**Authors:** Ann-Christin Pfeifer, Dorothee Amelung, Carina Gerigk, Corinna Schroeter, Johannes Ehrenthal, Eva Neubauer, Marcus Schiltenwolf

**Affiliations:** Center for Orthopedics, Trauma Surgery and Spinal Cord Injury, Heidelberg University Hospital, Schlierbacher Landstr 200a, 69118 Heidelberg, Germany; University of Heidelberg, Grabengasse 1, 69117 Heidelberg, Germany; Department of General Internal Medicine and Psychosomatics, Heidelberg University Hospital, Thibautstr 2, 69115 Heidelberg, Germany; Department of Orthopedics, Outpatient Multidisciplinary Pain Clinic, Trauma Surgery and Paraplegiology, Heidelberg University Hospital, Schlierbacher Landstr. 200a, 69118 Heidelberg, Germany

**Keywords:** Attachment-based intervention, Multimodal pain therapy, Chronic pain

## Abstract

**Background:**

The concept of attachment is relevant for the onset and development of chronic pain. Insecure attachment styles negatively affect therapeutic outcome. Insecurely attached patients seem to be less able to sustain positive effects of a multimodal treatment program. However, it has never been tested before if an attachment-oriented approach can improve treatment results of insecurely attached patients in a multimodal outpatient setting. To test this assumption, we compare the short- and long-term outcomes for pain patients who will receive multidisciplinary, attachment-oriented treatment with the outcomes for patients in a control group, who will receive the multidisciplinary state-of-the-art treatment.

**Methods:**

Two patient groups (baseline, attachment intervention) are assessed before treatment, after treatment, and at a 6 month follow-up. The study is conducted in a block design: After data collection of the first block (controls) and before as well as during data collection for the second block (treatment group), the health care personnel of the outpatient pain clinic receives training on attachment theory and its use in the therapeutic context. Pain intensity as measured with visual analogue scales and physical functioning will serve as the primary outcome measures.

**Discussion:**

The design of our study allows for a continuous exchange of experienced team members, which may help bring about concrete attachment related guidelines for the enhancement of therapeutic outcome. This would be the first attempt at an attachment-oriented improvement of multimodal pain programs.

**Conclusion:**

An attachment-based approach may be a promising way to enhance long-term treatment outcomes for insecurely attached pain patients.

**Trial registration:**

DRKS00008715 (registered on the 3^rd^ of June 2015).

## Background

Today, it is widely acknowledged that the onset and development of chronic pain syndromes is a result of complex interactions between biological, psychological and social influences, including patients’ beliefs about their self-efficacy, hypervigilant monitoring of bodily sensations, familial conflict or social support [19, 21, 22, 32]. If patients, for example, exhibit maladaptive behavioral or cognitive responses to acute episodes of pain, the pain may become chronic, thus affecting the long-term course [41].

**Fig. 1:**
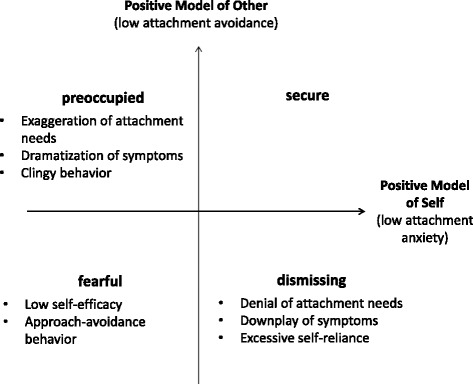
Attachment Model

Attachment theory provides a useful framework to classify patients’ relatively stable cognitive, emotional and behavioral response styles to stressors (such as pain), which have been successfully linked to disease processes in general [24, 35] and to diagnosis and processes of pain-related diseases in particular [37, 43]. Based on their dominant response patterns, adults can be classified to one of four attachment styles - one *secure* style, and the three insecure styles *dismissing*, *preoccupied*, and *fearful* [2, 38]. Bartholomew and Horowitz developed a model based on these four attachment styles (for a simplified overview, see Fig. 1). This model shows that adults with a secure style see themselves and others in a positive way, whereas fearful adults tend to see themselves and others in a negative way [2].

These attachment styles relate to patients’ stress responsiveness, their beliefs about their ability to cope with the experience of pain, or specific interaction patterns with spouses or health care personnel [14, 39]. Patients with insecure attachment patterns report higher levels of pain [36, 51], higher degrees of disability [12], lower levels of pain efficacy [39], show less functional and more dysfunctional coping strategies such as catastrophizing [10], and greater levels of depression and anxiety [1, 40]. Bartholomew and Horowitz [2] also demonstrated that secure and dismissing individuals report higher measures of self-esteem on Rosenberg’s Scale [44] compared to fearful and preoccupied individuals. Previous findings suggest that attachment security can be associated with higher self-esteem, whereas, low self-esteem seems to be more related to insecure attachment patterns [5, 11, 18].

These findings are especially relevant given that insecure attachment styles are overrepresented in patients suffering from chronic pain [12, 46]. Attachment insecurity also negatively influences the working alliance with health-care professionals [4], and outcome in psychosocial interventions [31].

**Fig. 2:**
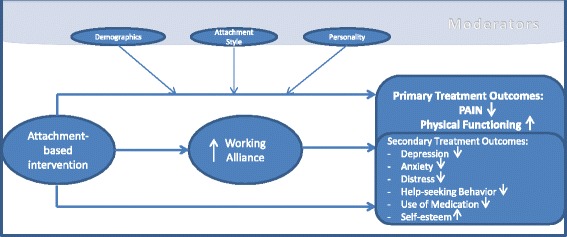
Attachment-based Working Alliance Model

To date, the knowledge about attachment concepts has not been used to improve pain treatment outcomes with the help of a differential approach based on individual attachment styles. Therefore, we will compare the short- and long-term treatment outcomes for pain patients who will receive multidisciplinary, attachment-specific treatment with the outcomes for patients in a control group who will receive the multidisciplinary state-of-the-art treatment (for a simplified overview, see Fig. 2). Our preliminary hypotheses were formulated based on the abovementioned information.

1. Patients in the intervention group, who receive an attachment-based multidisciplinary treatment, will report a larger mean reduction of the pain intensity between pre-treatment and post-treatment assessments than patients in the control group, who receive the state-of-the-art multidisciplinary treatment. It is expected that this larger reduction of the pain intensity in the intervention group compared to controls can be specifically attributed to better outcomes for the intervention group patients than control group patients who have been assessed with insecure attachment styles. Moreover, patients with an insecure attachment style and patients with more attachment anxiety are expected to report higher levels of pain intensity as well as physical functioning.
2. The intervention is specifically designed to improve the therapeutic relationship and to hereby improve treatment outcomes. Therefore, we expect to find improved ratings of the relationship (working alliance) between patients and therapists in the intervention group which will mediate the treatment outcomes pain intensity and physical functioning.
3. There will be a greater decline in help-seeking behavior and use of medication in the intervention group compared to the control group from pre-treatment to after the 6-month follow-up. This will be especially hypothesized for insecurely attached patients in the intervention group.
4. Subjective self-awareness of the patients particularly with regard to self-esteem will be expected to change to a greater extent in the intervention group compared to the control group.

## Methods

### Design

The study is conducted in a block design with two patient groups as blocks (baseline, attachment intervention) and 3 assessments T1, T2, and T3 (before treatment, post-treatment, and at a 6 month follow-up). We decided against a randomized controlled trial because (a) we had ethical concerns with a design that would put patients on a waiting list for several months, and (b) a block design ensures that the intervention’s effects only influence the outcomes of the treatment group: After collection of data for the control group, the health care personnel of the outpatient pain clinic receives training sessions on attachment theory and its use in the therapeutic context. These training sessions will not be carried out until data collection for the control group will be complete. More attachment-related training sessions alternating with supervision meetings will be held on a monthly basis to assist the therapeutic team in the practical application during data collection for the second sample, the treatment group. During our weekly team meetings, the case reviews will be complemented with a discussion on the individual attachment style of each patient. Also these meetings will be used to collect situations, which are perceived to be critical for the forming of a working alliance by the therapeutic team (for example, situations in which the patient misses entire therapeutic sessions or appears too late to them on a regular basis). These critical situations will be subsequently used to structure the discussions of individual cases in the bi-monthly training sessions.

### Participants

#### Inclusion and exclusion criteria

Our participants are enrolled as outpatients in the orthopedic clinic of the Heidelberg University Hospital and participate in a four-week outpatient multidisciplinary pain treatment including physiotherapy, ergotherapy, music and dance therapy, individual and group psychotherapy. As such, they

- have experienced chronic pain for at least six months
- are between 18 and 80 years of age
- have previously received standard treatment consisting of at least one rehabilitation program or two inpatient treatments, which did not yield lasting effects.

Exclusion criteria include

- high C-reactive protein (CRP) levels as an indicator of rheumatoid arthritis
- acute inflammations of the spine
- a tumor
- a diagnosed psychosis
- a diagnosis of a bipolar or neurological disorder
- an insufficient ability to communicate in German

### Ethics statement

The study procedures were approved by the Institutional Review Board of the Medical Faculty, University of Heidelberg (S-305/2013). As such, they comply with the ethical standards established in the Declaration of Helsinki and with the guidelines of ICH-GCP. Upon arrival at the department, the principal investigator verbally states the study purpose and procedures to eligible patients and informs them about their right of withdrawal at all times. Additionally, all participants obtain an information sheet on the study objectives and modalities, data preparation and pseudonymized data storage. All participating patients provide written informed consent.

### Sample size calculation

We calculated the necessary sample size for each of our two subsamples (intervention and control) with the G*Power Analysis software program for two-sample t-tests [17], and by reference to our primary outcome measure (pain intensity). Based on the results of our pretests, we expected a small to medium effect of 0.4 for the comparison of the two groups (with an alpha level = 0.05, two tailed, β = 0.2). Results indicate that at least 93 participants are required for each group, for a total of 186.

### Statistical analysis

Descriptive statistics will be used to describe the characteristics of the study sample. Analysis of variance will be used for the evaluation of the changes of the continuous outcomes pain intensity and physical functioning (T1-T2, T1- T3). Research condition (attachment style and study group), will be used as independent variable. Demographic variables such as sex, age, education and clinical variables such as opioid intake and comorbidity will be entered as covariates. Partial regression coefficients will be used to determine if the “working alliance” mediates the effect of attachment on the continuous outcomes (pain and physical functioning).

Missing data on the questionnaires will be handled according to the questionnaire protocol.

### Intervention

By drawing on attachment theoretical ideas, we do not mean to change any of the overall aims of multimodal pain treatment. Also, it would be unrealistic to aim at a complete reversal of underlying insecure attachment patterns to a secure pattern in such a setting.

Rather, we aim at an improvement of the therapeutic alliance (a) in terms of the therapists’ general ability to provide a secure base for his patients, and (b) in the therapists’ ability to understand and deal with their patients’ individual attachment-based motivations and needs. Thus, the intervention’s guidelines should help facilitate the attainment of the program’s aims by enhancing the patient’s sense of having a secure base. Therefore, our intervention training includes both general directions for building a meaningful therapeutic relationship, and guidelines, which are specifically tailored to the needs of individual attachment styles.

To develop general guidelines for an improvement of the working alliance, we identified as useful starting point the therapeutic approach of the complementary therapeutic relationship. This approach emphasizes the underlying motives (such as the attachment motive) of patients [9, 48].

We also developed more specific guidelines for each attachment style based on a further development of existing literature on the application of attachment ideas to specific therapeutic settings. For example, attachment theoretical ideas have been incorporated in therapeutic programs for the treatment of borderline personality disorder, depression, medically unexplained symptoms, family and couple therapy [3, 13, 25, 28, 29]. A multimodal rehabilitation program for children and adolescents with functional impairment in Australia applies for instance attachment principles to their family-based intervention [29, 30]. However, as guidelines often remain vague, are tailored to one specific therapeutic school or are given on the assumption of a long-term therapy, all of which is not suitable for our context, we only used them as a starting point.

For example, patients with preoccupied attachment styles might benefit more from an initially concordant approach which puts emphasis on the therapist’s role as a secure base. Patients with preoccupied or fearful attachment styles might feel overwhelmed by a program which is too fast in emphasizing autonomy, and possibly reinforce existing fears of rejection and abandonment. On the other hand, dismissively attached patients might feel uncomfortable with high levels of proximity, and the amount of guidance and care preoccupied patients favor to feel safe.

The multimodal setting requires an attachment-based concept, which can be employed by medical personnel with diverse professional backgrounds such as doctors, physiotherapists, occupational therapists, music and dance therapists. Additionally, therapists may avail themselves of diverse methodological approaches such as psychoanalytical or cognitive behavioral therapy.

Each team member is provided with a manual of the general guidelines and the attachment style-specific guidelines. In the course of data collection for the intervention group, these guidelines will also be constantly extended.

### Measures

#### Psychopathology

The Structured Clinical Interview for DSM-IV Axis I Disorders (SCID-I; [54]) is a semi-structured interview for assessing DSM-IV Axis I diagnoses. It is considered diagnostic gold standard in psychiatric assessment, and has shown high reliability and superior validity when conducted by trained health professionals.

#### Demographic information, help-seeking behavior and use of medication

Items are adapted from the German Pain Questionnaire. The German pain questionnaire (DSF; [42]) has been developed and validated by the International Association for the Study of Pain (DGSS). The concept of the DSF is based on a bio - psycho - social pain model. The modular approach to pain assessment consists of: demographic data, pain variables (e. g. pain sites, temporal characteristics, duration, intensity), pain associated symptoms, affective and sensory qualities of pain (adjective list by Geissner, SES Copyright, [23]), pain relieving and intensifying factors, previous pain treatment procedures, pain-related disability (Pain Disability Index by [49]), depression test CES-D (Center for Epidemiological Studies Depression Test), comorbid conditions, social factors (educational level, occupation, retirement status, compensation and/or litigation status, disability for work),health related quality of life (SF-36 Copyright).

#### Attachment style

Assessments of both intervention group and baseline group are identical. For an overview of all measures at T1, T2, and T3, see Table 1. Patients’ attachment patterns will be assessed with the help of the relationship questionnaire (RQ 2; [2]) and the revised Experience of Close Relationships questionnaire [15] in its newly developed German short version [6, 15] . The ECR-RD 12 has been anchored in two different ways to assess the two regulatory attachment strategies *avoidance* and *anxiety* (a) specifically with regard to partners or spouses and (b) more globally with regard to friends and relatives. The original version of the ECR-R [20] was based on the principles of item response theory and specifically assesses attachment representations in romantic relationships (e.g. attachment anxiety: ‘I find that my partner/partners doesn’t/don’t want to get as close as I would like’; attachment avoidance: ‘I find it difficult to allow myself to depend on romantic partners’).

**Table 1:** Overview of variables and instruments

Domain	Instruments	T1	T2	T3
Psychological disorders, axis 1	*German semi-structured Clinical Interview* based on DSM-IV (SKID-I)	**×**		
Socio-demographic variables	Items adapted from the *German Pain Questionnaire*	**×**		
Help-seeking behavior	Items adapted from the *German Pain Questionnaire*	**×**		**×**
Use of Medication	Items adapted from the *German Pain Questionnaire*	**×**		**×**
Attachment Patterns	•* Relationship Questionnaire* (RQ 2) •* Experience of Close Relationships* (ECR-RD 12) with regard to a) partner/spouse b) friends/relatives	**×**		**×**
Pain	•* Pain Intensity* **:** Visual Analogue Scales (VAS) • *Pain Frequency*: number of days with pain, number of days with strong pain (previous month)	**×**	**×**	**×**
Physical Functioning	• *Oswestry Disability Index* (ODI) •* Health Survey* (SF12)	**×**	**×**	**×**
Emotional Distress	•* Depression, Anxiety and Stress Scale* (DASS) •* Health Survey* (SF12)	**×**	**×**	**×**
Interpersonal Problems	*Inventory of Interpersonal Problems* (IIP 32)	**×**	**×**	**×**
Self-esteem	*Rosenberg Self Esteem Scale* (RSES)	**×**	**×**	**×**
Coping	*Coping Strategies Questionnaire* (CSQ-D)	**×**	**×**	**×**
Evaluation of Therapeutic Process	•* Working Alliance Inventory* (WAI) •* Therapie Stations-Erfahrungs-Bogen* (TSEB)		**×**	

*T1, before treatment; T2, after treatment; T3, six months follow-up*

The ECR-RD has shown high levels of test-retest stability and discriminant validity [47]. We decided to include both an assessment of attachment styles and attachment dimensions to enhance measurement precision and hereby facilitate the interpretation of results. The brief self-report RQ 2 captures a person’s dominant (cognitive) schematic representation of self and others. It consists of four short paragraphs, each describing one attachment pattern (e.g. the fearful prototype reads, ‘I am uncomfortable getting close to others. I want emotionally close relationships, but I find it difficult to trust others completely, or to depend on them. I worry that I will be hurt if I allow myself to become too close to others’). The participants is then asked to rate their degree of correspondence to each prototype on a 7-point Likert-type scale ranging from 1 (completely true) to 7 (completely false). The therapeutic team will be informed about the results only in the treatment condition. In this way, treatment can be tailored to each patient’s specific attachment needs.

From these four prototype ratings, the ‘model of self’ and the ‘model of other’ scales were calculated as proposed by the authors. Patients with positive models of both self and other were classified as ‘secure’, patients with negative/neutral models for both as ‘fearful’, and individuals with mixed results as ‘preoccupied’ or ‘dismissing’. The RQ-2 has been shown to have an acceptable level of psychometric soundness as a brief screening instrument, and it is relatively independent from self-deceptive biases. The RQ-2 implemented has been used in multiple international studies and proved to be an efficient screening instrument with good construct validity across cultures.

### Primary outcome measures

With two Visual Analogue Scales, we assess the pain intensity at present and within the previous week. Patients are also asked to indicate the number of days they experienced pain, and the number of days they experienced strong pain, both within the previous month. Physical functioning will be assessed with the Oswestry Disability Index by Mannion et al. [34], which has been specifically developed for use with pain patients. Also, we included a widely used German short version of the Health Survey to assess physical functioning and health-related quality of life more generally [8].

#### Pain intensity and functional impairment

Subjective pain intensity over the preceding week was measured on a visual analogue scale (VAS; [26]) ranging from 0 (no pain) to 100 (worst imaginable pain). Each patient indicated a position on the VAS in response to the question “How severe was your pain on average in the last seven days?” The VAS is a valid and reliable instrument for measuring the intensity of pain.

#### Physical functioning

The validated Oswestry Disability Index (ODI; [16]) is currently considered by many as the gold standard for measuring degree of disability and estimating quality of life in a person with low back pain. The self-completed questionnaire contains ten topics concerning intensity of pain, lifting, ability to care for oneself, ability to walk, ability to sit, sexual function, ability to stand, social life, sleep quality, and ability to travel.

#### Emotional distress

The Depression Anxiety Stress Scales (DASS; [33]) is made up of 42 self-report items, each reflecting a negative emotional symptom. Each of these is rated on a four-point Likert scale of frequency or severity of the participants' experiences over the last week with the intention of emphasising states over traits. These scores ranged from 0, meaning that the client believed the item "did not apply to them at all", to 3 meaning that the client considered the item to "apply to them very much, or most of the time". It is also stressed in the instructions that there are no right or wrong answers. The main purpose of the DASS is to isolate and identify aspects of emotional disturbance; for example, to assess the degree of severity of the core symptoms of depression, anxiety or stress.

The SF-12 Health Survey [7] is a shorter version of the SF-36 Health Survey that uses just 12 questions to measure functional health and well-being from the patient’s point of view. Taking only two to three minutes to complete, the SF-12 is a practical, reliable and valid measure of physical and mental health and is particularly useful in large population health surveys or for applications that combine a generic and disease-specific health survey.

#### Interpersonal problems and self-esteem

The inventory of Interpersonal Problems (IIP 32; [27, 50]) is a self-report instrument that identifies a person's most salient interpersonal difficulties. The items provide representative interpersonal problems that are commonly reported in initial interviews.

The Rosenberg Self-Esteem Scale [44], a widely used self-report instrument for evaluating individual self-esteem, was investigated using item response theory. Factor analysis identified a single common factor, contrary to some previous studies that extracted separate Self-Confidence and Self-Depreciation factors. A 10-item scale that measures global self-worth by measuring both positive andnegative feelings about the self. The scale is believed to be uni-dimensional. All items are answered using a 4-point Likert scale format ranging from strongly agree to strongly disagree.

#### Coping

Coping strategies in the context of chronic pain refer to the way individuals who experience pain develop ways to tolerate, minimize or reduce their pain. The Coping Strategies Questionnaire (CSQ; [52]) is internationally the most widely used measure of pain coping strategies. The value of the CSQ is its ability to assess various cognitive and behavioral coping factors derived from a rationally constructed pool of strategies reported by patients experiencing pain and cross-validated by pain clinicians and pain psychologists. It identifies both active and passive coping strategies.

#### Evaluation and therapeutic process

The Working Alliance Inventory-Short Revised (WAI-SR; [53]) is a recently refined measure of the therapeutic alliance that assesses three key aspects of the therapeutic alliance: (a) agreement on the tasks of therapy, (b) agreement on the goals of therapy and (c) development of an affective bond.

The German Stations-Erfahrungs-Bogen [45] is an instrument for the process evaluation inpatient psychotherapy. Application possibilities are in the clinical area, in science as well as in the quality monitoring of the treatment facility. The TSEB is a patient questionnaire for the assessment of experiencing inpatient psychotherapy. With 38 items, the self-assessment of patient regarding the following subscales is applicable: relationship to therapeutic team, relationship with the therapist, emotional atmosphere between the patient, need for affection by the other patients, experiencing the intensity of treatment, experience the therapeutic framework and self-efficacy (e.g. relationship with therapist: ‘I found it easy to connect with my therapist’).

## Discussion

### Potential strengths

Continuous exchange of experienced team members might help bring about concrete attachment related guidelines for the enhancement of therapeutic outcome for pain patients and allow us to develop a standardized training manual for pain patients. To our knowledge, such concrete guidelines are rare in applications of attachment concepts to therapy of chronic pain patients in a multimodal setting. Also, as patients with preoccupied and fearful attachment styles are less able to maintain the positive results of multimodal pain treatment over a period of time, an attachment-based approach is a promising way to enhance the prospects especially for these patients.

### Potential limitations

We do not believe that two training sessions for the therapeutic team will suffice to yield large effects on therapeutic outcome. However, we expect that the constant exchange within the team during weekly team meetings and monthly supervision sessions will facilitate the development of a clear understanding of attachment styles as well as a repertoire of methods to deal with incidents, which are critical for the working alliance. Therefore, therapists are likely to become more seasoned in recognizing and dealing with the attachment behavior of their patients in the course of the study. As a result, effects might only become apparent in an advanced phase of data collection for the intervention group.

Patients with a preoccupied attachment style and, even more pronounced, the ones with a fearful attachment style, gain the least from the state-of-the art multidisciplinary pain treatment. These patients are known to have difficulty with the regulation of distance and proximity to other people. Our program in the outpatient clinic offers many opportunities to express distancing or proximity-seeking behavior in dealing with doctors, physiotherapists, psychologists and the group of other patients and therefore provides many chances for difficulty. Even if the therapeutic team is trained in attachment concepts, the time frame might not be enough to use this new knowledge to their advantage. It is very likely that in such a setting, patients with insecure attachment patterns will at some point elicit (negative) reactions either from staff or from other patients similar to the ones they continuously experience in their every-day lives. This would confirm their general expectations of themselves and others in relationships, hereby reinforcing these expectations.

The short time frame of this setting, which is already packed with activity, might simply not be enough to adequately absorb these experiences and to tap into them in a therapeutic sense. Therefore, the setting in itself could be unsuited to a treatment concept which is individually tailored to specific attachment styles. It might even be unsuited altogether for patients who are known to have difficulty to become self-dependent once the intense support they receive throughout the program discontinues. On the other hand, as such an individually tailored attachment concept has never been applied to multimodal pain treatment and attachment styles clearly are very relevant in this setting, there is also the chance that treatment can be largely improved. Moreover, we do not aim at altering the individual attachment patterns of the patients themselves. Rather, we use the knowledge about these patterns to form an adequate basis from which we can start working with the patients on their pain related goals without overstraining them.

## Conclusions

### Trial status

**Fig. 3:**
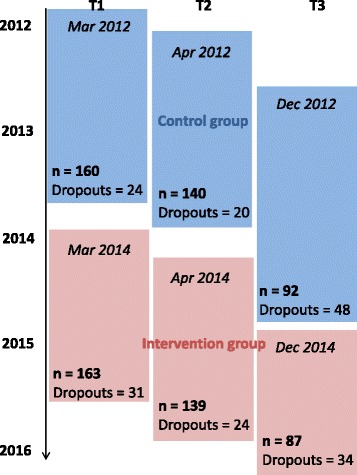
Dropouts

Recruitment of patients for the control group started in March 2012. Pre- and post-treatment assessments were complete by Oct. 2013 with a total of 184 patients who received a questionnaire at T1 (for an overview of the recruitment process including dropout numbers, see Fig. 3). The 6-month follow-up questionnaires of the control group were completely sent out by May 2014. Each team member received monthly intervention training sessions between Nov. 2013 and May 2015. For an overview of the received attachment training, see Table 2. Recruitment of patients for the intervention group started in March 2014. Pre- and post-tretments were complte by Dec. 2015 with a total of 194 patients who received T1. The 6-months follow-up questionnaires of the interventions group will be completely sent out by June 2016.

**Table 2:** Overview of the attachment training

	Title	Training
1	Attachment theory – An introduction	Development of attachment patterns and behavior
2	Attachment based intervention in the therapeutic treatment process	> therapist as secure base > guideline for patients with insecure attachment styles
3	Impact of attachment on rehabilitation from disease	Attachment theory: meaning in regards to psychodynamic rehabilitation
4	Basics of attachment theory in a clinical setting	„Secure base“- > How?
5	Attachment and pain	Treatment of chronic pain patients – development of an attachment based intervention
6	Mentalisation based therapy	How do I see myself? How do I see others? Patient should learn more about himself and the behavior of others
7	Collegial consulting	Handling of patients with different attachment styles
8	Self-compassion and attachment	Empathy for others - > What is empathy in the therapist-patient-relationship?
9	Self-awareness- Attachment experiences	> childhood memories > In adulthood: Do some relationship patterns repeat itself? > How to cope with stress?
10	Psychodynamic concepts	> unconscious conflicts > Introjection > transference and countertransference > dysfunctional relationship patterns
11	Personality traits - narcissism	> relationship patterns > appearance > therapy
12	Secure attachment style and psychodynamic therapy I (Jeremy Holmes Ph.D., University of Exeter, London)	„Attachment, psychotherapy, and the inner world: from ethology to rational neuroscience“
13	Secure attachment style and psychodynamic therapy II (Jeremy Holmes Ph.D., University of Exeter, London)	„Partially contingent mirroring: a key component of psychoanalytic meta-competence” - > application of attachment research findings to clinical situations
14	Interpersonal therapy- communication	Theory and examples of good and bad communication between therapist/doctor and patient
15	Attachment based intervention	> examples and advice for the therapist
16	Handling of critical situations	Patient’s behavior - > attachment style - > reaction of the therapist
17	Exposure	Motivation and psycho-education
18	Motivational and complementary therapeutic relationship according to Grawe	A basis for an attachment based approach?
19	Doctor-patient communication	Communication skills
20	Impact of personality and attachment style on pain perception in the therapeutic treatment process	Short and long term impact of attachment patterns on the treatment outcome

## Abbreviations

CRP: C-reactive protein test
GCP: Good clinical practice
RQ: Relationship questionnaire
ECR: Experience of close relationships questionnaire
VAS: Visual analogue scale
ODI: Oswestry disability index
SF12: Health survey
DASS: Depression, anxiety and stress scale
IIP: Inventory of interpersonal problems
RSES: Rosenberg self esteem scale
CSQ-D: Coping strategies questionnaire- German version
WAI: Working alliance inventory
TSEB: Therapie Stations-Erfahrungs-Bogen
